# A Polycationic Brush Mediated Co-Delivery of Doxorubicin and Gene for Combination Therapy

**DOI:** 10.3390/polym11010060

**Published:** 2019-01-03

**Authors:** Wenjuan Chen, Mingming Zhang, Wei Shen, Bo Du, Jing Yang, Qiqing Zhang

**Affiliations:** 1 Tianjin Key Laboratory of Biomedical Materials, Institute of Biomedical Engineering, Chinese Academy of Medical Sciences & Peking Union Medical College, Tianjin 300192, China; chenwenjuanvv@163.com (W.C.); sw895971588@163.com (W.S.); dubojlu@163.com (B.D.); yangjing37@hotmail.com (J.Y.); 2 Institute of Biomedical and Pharmaceutical Technology, Fuzhou University, Fuzhou 350002, China

**Keywords:** polycationic brush, co-delivery system, cyclodextrin polymer, combination therapy

## Abstract

The combination of drug and gene strategies for cancer therapy, has exhibited greater effectiveness than drug or gene therapy alone. In this paper, a coil-comb shaped polycationic brush was used as a multifunctional carrier for co-delivery of drug and gene. The side chains of the comb block of the brush were composed of cyclodextrin (CD)-containing cationic star polymers, with a super-high density of positive charge. Doxorubicin (DOX) could be loaded into the cavity of CD polymers to form DOX-loaded nanoparticles (DOX-NPs) and the p53 gene could be subsequently condensed by DOX-NPs. The obtained DOX-NPs/pDNA complexes were less than 150 nm in size, and so could transport DOX and the gene into the same cell. The complexes performed well with regards to their transfection efficiency on MCF-7 cancer cells. As a result, enhanced cell growth inhibition, with decreased DOX dosage was achieved due to the synergistic effect of co-delivery of DOX and the p53 gene. This finding provides an efficient approach for the development of a co-delivery system in combination therapy.

## 1. Introduction

Co-delivery of biomolecules has wide applications in clinical diagnosis and treatment. For example, co-delivery of antigens and immune-stimulators can be more effective in activating immune responses and is of great value in cancer immunotherapy [1]. Moreover, co-delivery of contrast reagents and anti-tumor drugs can achieve cancer theranostics [2]. For gene therapy, combining drugs and genes strategies for cancer treatments has exhibited greater effectiveness compared with drug or gene therapy alone [3,4,5,6,7]. Due to the synergistic effect of the co-delivered drug and gene, decreased drug dosage, negligible side effects and high inhibition efficacy can be achieved [8,9]. Moreover, co-delivery of anti-tumor drugs and genes can also effectively prevent multidrug resistance of tumor cells [10]. The key point of synergistic therapy is to deliver drug and gene to the same cell and tissue. As a result, there has been increasing interest around the development of multifunctional delivery systems based on liposomes [11], cationic micelles [12,13,14], dendrimers [15], and inorganic nanoparticles [16]. Cyclodextrin (CD)-based polymers have been widely used in drug delivery systems since they provide the cavities for binding hydrophobic drugs. In gene delivery systems [17,18,19,20,21], CD mediated polycations are also able to enhance gene transfection efficiency due to CD features such as molecular inclusion, membrane disturbing and macromolecule shielding [22,23,24]. Combining the above advantages, co-delivery systems based on CD polymers have been developed. For example, cationic β-cyclodextrin-polyethylenimine-DOX (PC-DOX) conjugates for carrying a p53-encoding plasmid, promoted the inhibition of tumor growth in vivo and prolonged the survival time of tumor-bearing mice [25]. A bioreducible CD polymer system for co-delivery of paclitaxel and a p53 gene induced efficient cell apoptosis [26].

The essential challenge for gene carrier design is to achieve a high gene transfection efficiency. Studies showed that short-chain and hyperbranched polycations exhibited higher transfection efficiency and lower cytotoxicity, compared with linear ones with similar molecular weight [27,28]. Polymer brushes are a such kind of material, with ultra-high-density chains. They always stretch out from the surface because the distance between neighboring chains is lower than two-fold the gyration radius of a free polymer chain [29]. When polymerized using cationic monomers, the polycationic brushes usually exhibited excellent condensing capability compared to the anionic polymers, such as DNAs, proteins, etc. The colloidal stability of the formed particles was also enhanced due to the high osmotic repulsions between the brush chains. As a result, they exhibited higher transfection efficiency and lower cytotoxicities when used as gene carriers [30,31].

**Scheme 1:**
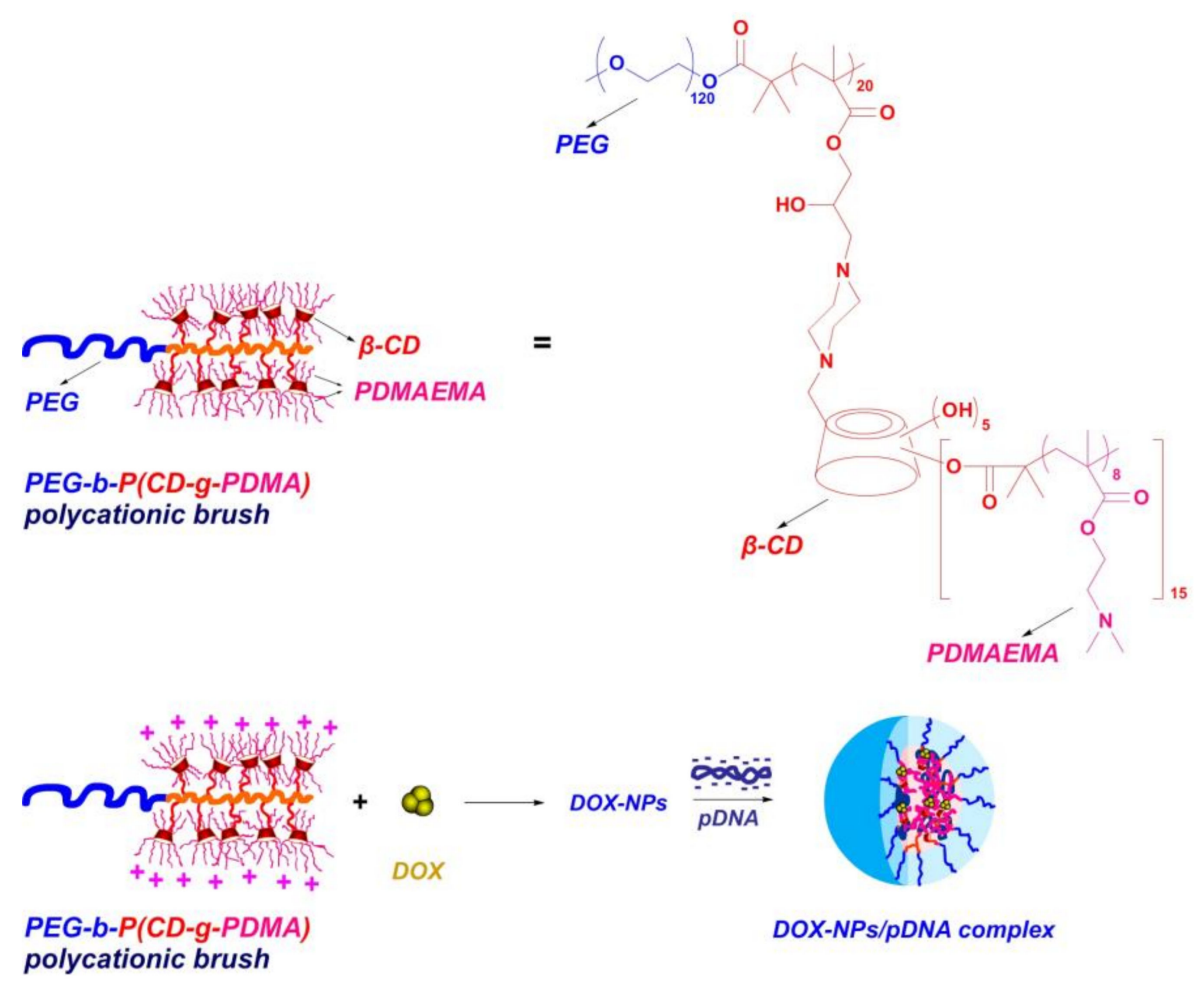
Chemical structure of the polycationic brush, preparation of doxorubicin-loaded nanoparticles (DOX-NPs) and multifunctional DOX-NPs/pDNA complexes.

In our previous work [32], a well-defined coil-comb shaped polycationic brush with “CD-containing cationic star polymers” as side chains, was synthesized by atom transfer radical polymerization (Scheme 1). Benefiting from both brush-like structures and CD-based star polycations, the brush exhibited significantly enhanced gene transfection efficiency than the relevant single star polycation or PEI25K in normal cells (COS-7 and 293T cells). Considering that CD can further be used as a host to bind hydrophobic drugs via inclusion interactions, we attempted to use this brush to construct a multifunctional carrier for co-delivery of doxorubicin (DOX) and a p53-encoding plasmid (Scheme 1). Such nanocomplexes were employed for inducing an efficient cancer cell death by the synergistic effect of DOX and the expressed p53 protein.

## 2. Results and Discussion

### 2.1. Preparation of Polycationic Brush and Characterizatioin

The polycationic brush was prepared according to our previous work [32]. In order to bind DOX more effectively, the degree of polymerization (DP_n_) of the CD monomer was increased to 20 in this study. Confirmed by the ^1^H NMR result (Figure S1), the chain number of cationic poly(2-dimethylaminoethyl methacrylate) (PDMAEMA) was 15 per CD and the DP_n_ of the DMAEMA monomer was 8 per chain. Thus, the molecular weight of the brush was calculated at about 423,600, according to the above DP_n_. The molecular weight distribution was then measured as 1.26 by gel permeation chromatography (GPC) (Figure S2).

### 2.2. Preparation of DOX-NPs and Drug Release Behavior

Driven by host–guest interactions between CDs on the brush and the hydrophobic DOX, DOX-loaded nanoparticles (DOX-NPs) were prepared. To obtain DOX-NPs with suitable physicochemical properties, different drug/polymer feed ratios were investigated and the characteristics of the nanoparticles are presented in Table 1. Both the loading content (LC) and the encapsulation efficiency (EE) increased with increasing drug/polymer feed ratio from 2/10 to 5/10. Attributed to the shielding effect of the PEG outer corona, zeta potential values for all DOX-NPs fluctuated around zero (less than 10 mV). The hydrodynamic diameters (D_h_s) of all the samples were below 200 nm, measured by dynamic laser scattering (DLS), demonstrating that they were appropriate for circulating in the body. However, only samples with a feed ratio of 2/10 and 3/10 showed relatively narrow size distribution. For further use, the DOX-NP with a drug/polymer feed ratio of 3/10 was selected as a representative formulation.

**Figure 1:**
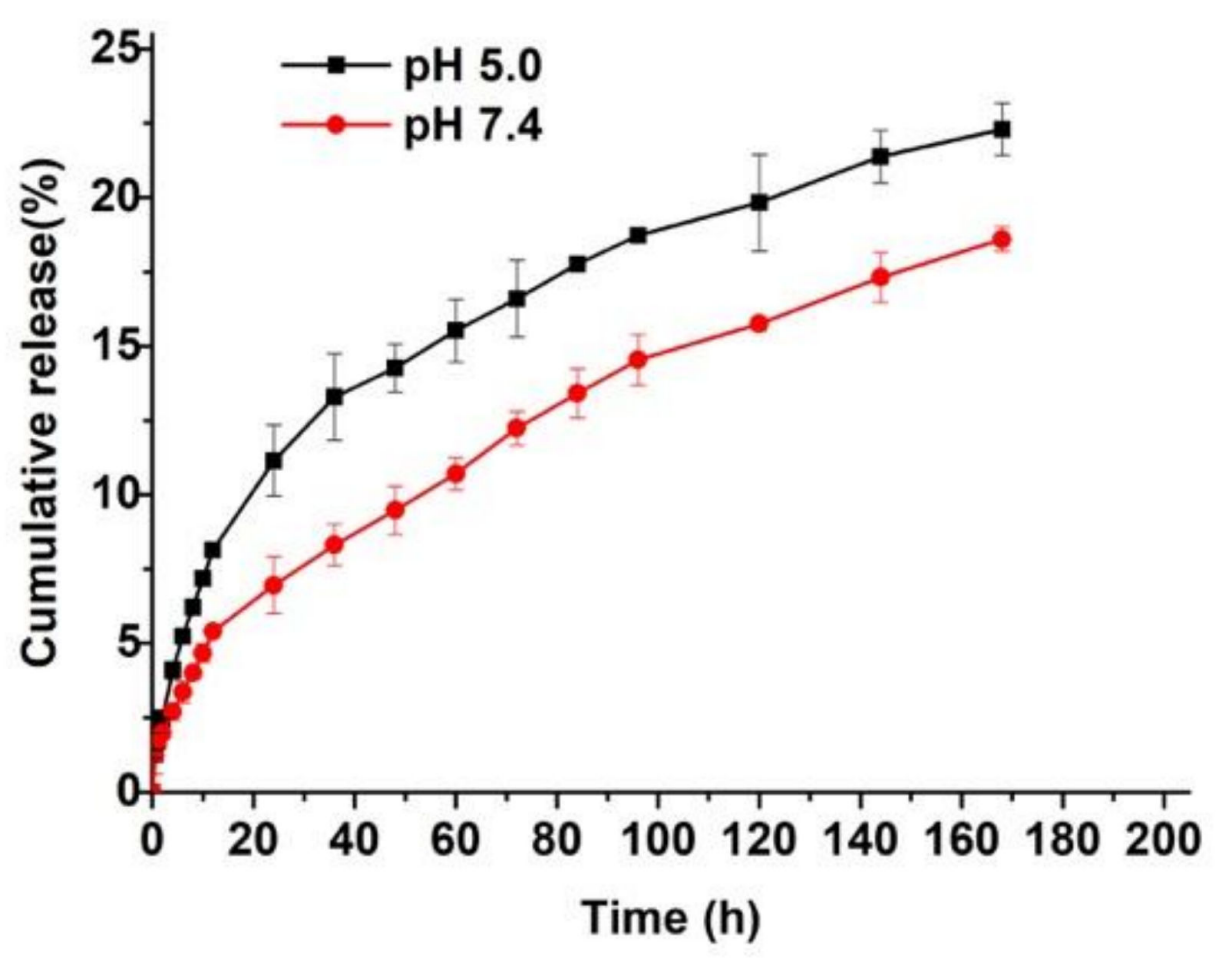
In vitro release profiles of DOX-NPs in PBS solution (pH 7.4) and acetate buffer saline (ABS) solution (pH 5.0), respectively.

In vitro release of DOX from DOX-NPs was then investigated in phosphate buffer saline (PBS, pH 7.4) and acetate buffer saline (ABS, pH 5.0) solutions, respectively. As shown in Figure 1, the DOX release rate was faster at pH 5.0 than 7.4. This is attributed to the following two factors. Firstly, the drug release rate was accelerated by the higher solubility of DOX in acidic solution than in neutral solution; secondly, DOX could be released more easily from the core due to the extension of the protonated PDMAEMA chains in the acidic condition. This is because PDMAEMA chains are pH-sensitive with a pK_a_ of about 7.1 [33]. This result indicates that DOX could be readily released via endosomal- (pH 5.5–6) or lysosomal-pH (pH 4–5) in cells [34] and thus could induce antitumor activity.

### 2.3. pDNA Condensation and Characterization

**Figure 2:**
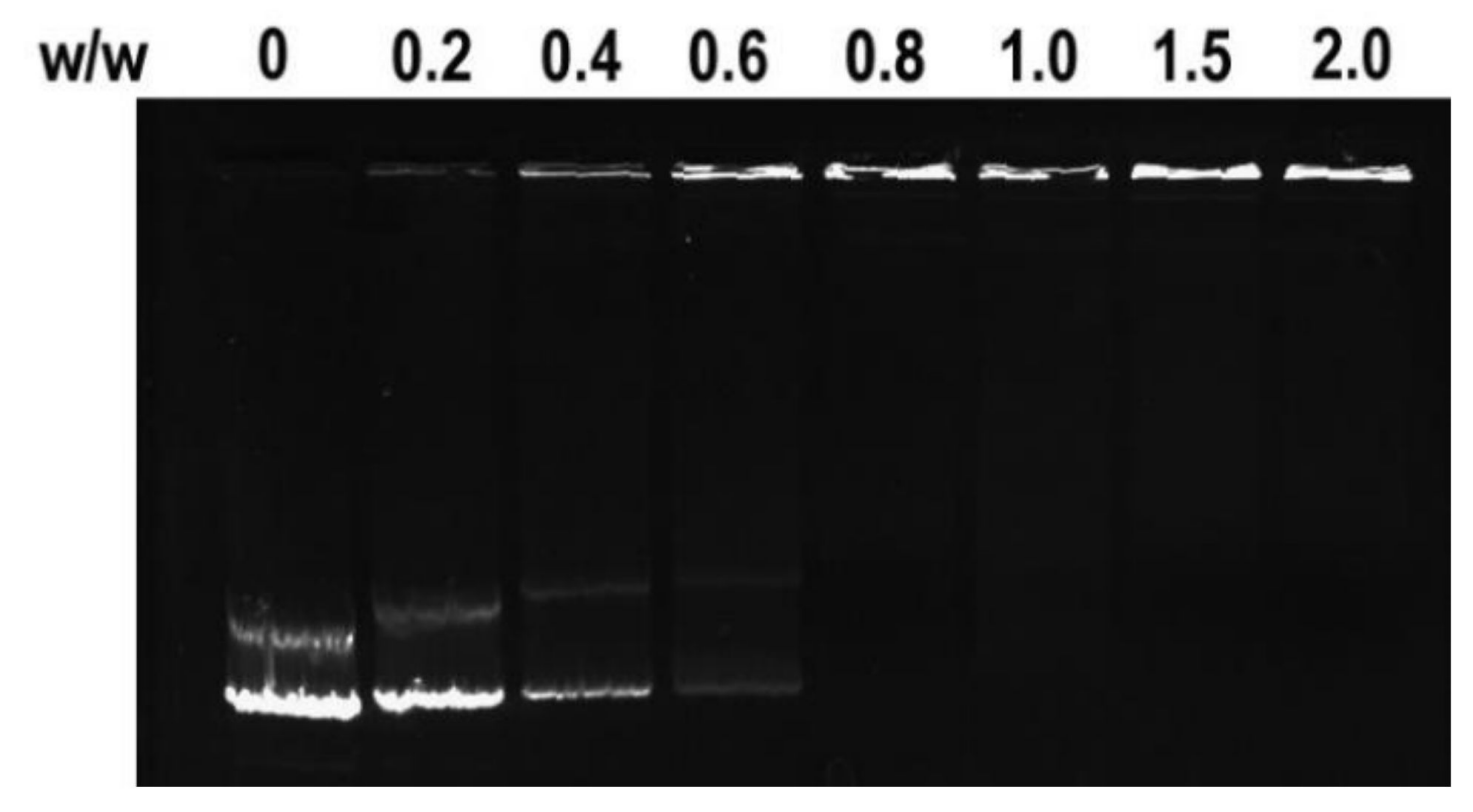
Agarose gel electrophoresis retardation assays of DOX-NPs/pDNA complexes at different *w*/*w* ratios.

**Figure 3:**
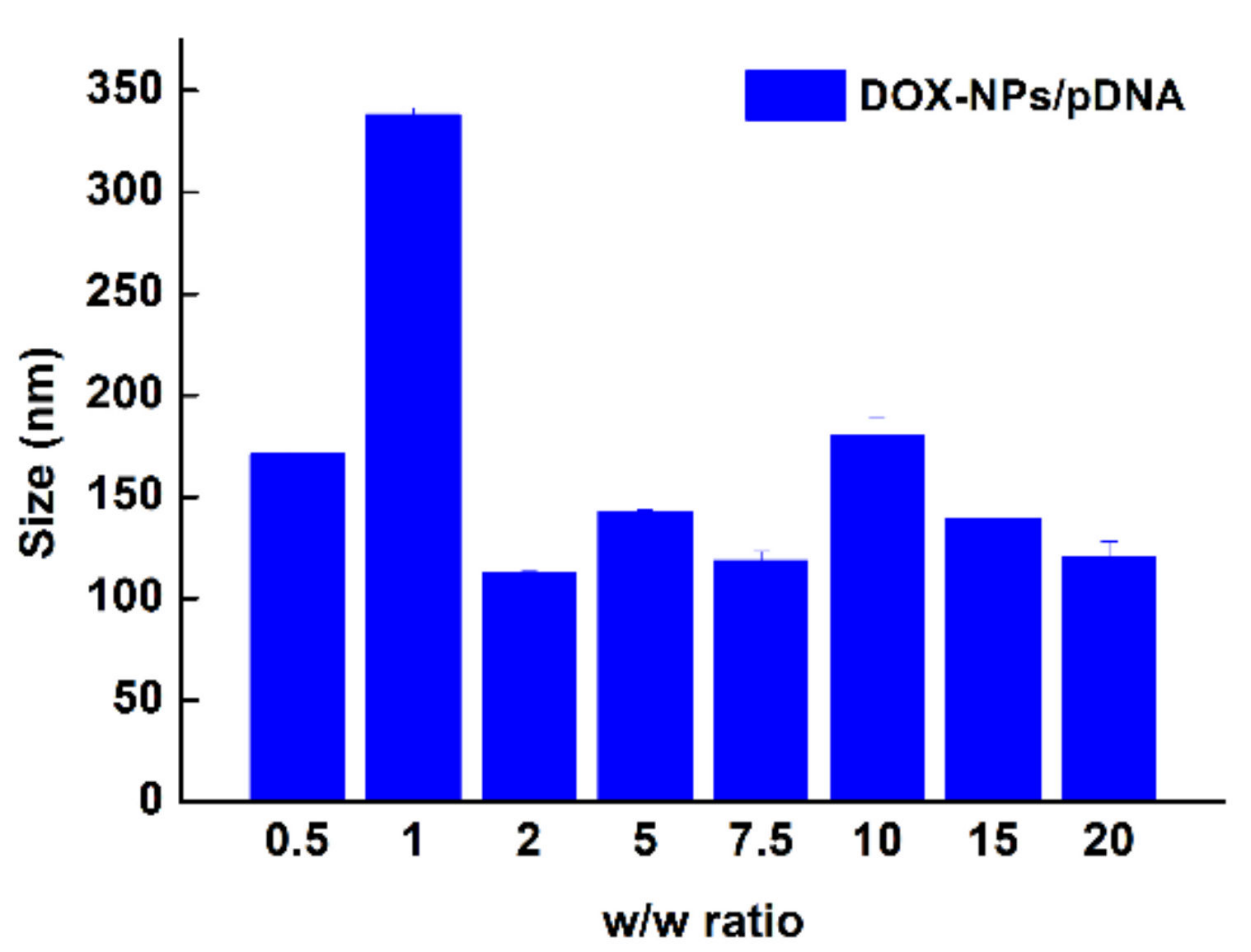
Particle size of DOX-NPs/pDNA complexes at different *w*/*w* ratios.

**Figure 4:**
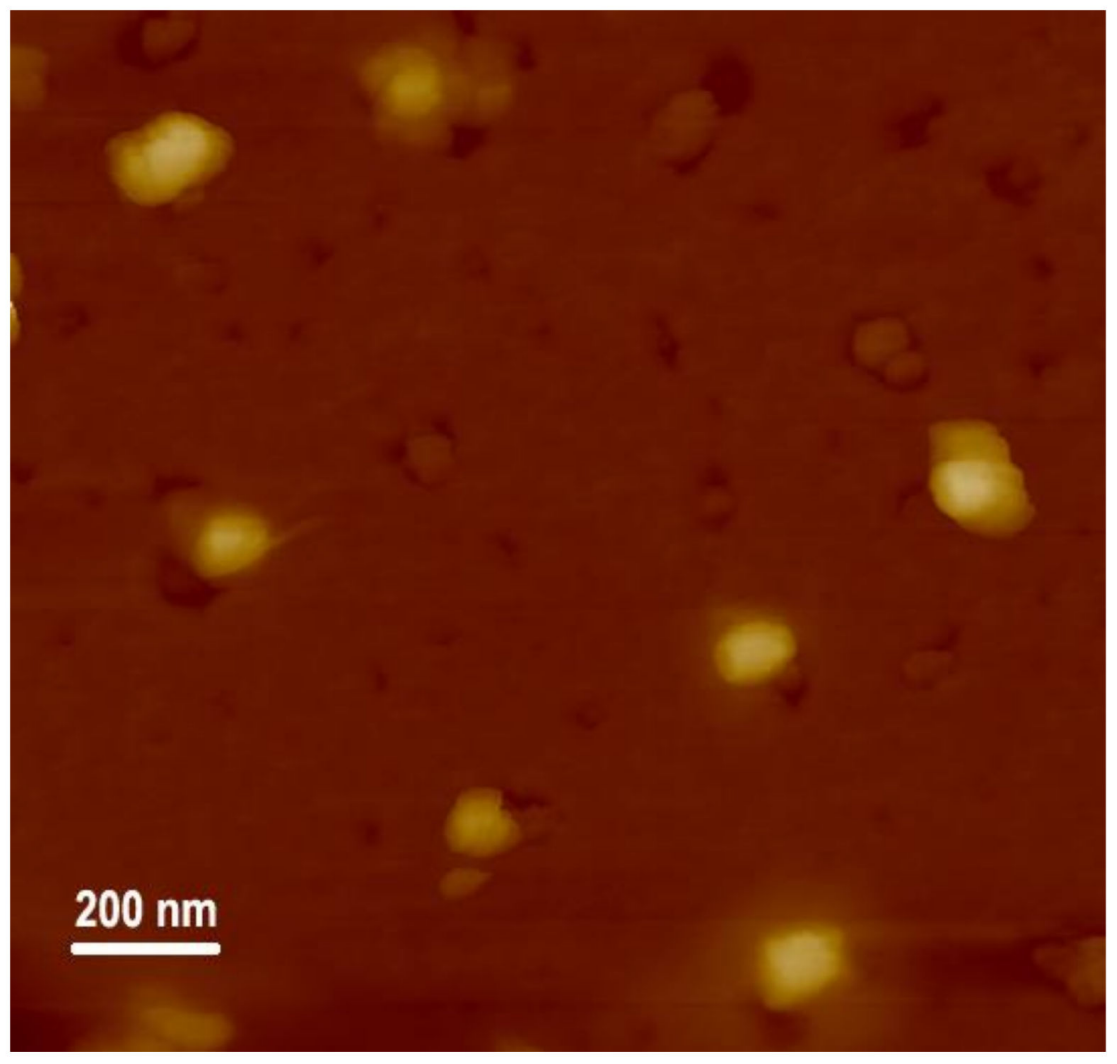
AFM image of DOX-NPs/pDNA complexes at optimal *w*/*w* ratio of 10.

Next, a multifunctional delivery system was constructed using DOX-NPs as carriers to condense pDNA. The binding ability of DOX-NPs to pDNA was analyzed by agarose gel electrophoresis. Results in Figure 2 show that DOX-NPs entirely inhibited pDNA migration when the *w*/*w* ratio was larger than 0.8. This demonstrates DOX-NPs exhibit good ability at condensing pDNA to form stable multifunctional complexes. The sizes of the complexes at various *w*/*w* ratios were then measured by DLS. As shown in Figure 3, when the *w*/*w* ratio is greater than 1, DOX-NPs efficiently compacted pDNA to form complexes with sizes less than 150 nm, indicating they were able to deliver pDNA into cells. Typical AFM images of DOX-NPs/pDNA complexes (*w*/*w* ratio of 10) showed that the complexes had an irregular spherical shape with a size less than 200 nm (Figure 4). This was smaller to the size of naked pDNA [35], indicating that pDNA was tightly condensed by the DOX-NPs.

### 2.4. Co-Localization of Drug and Gene

**Figure 5:**
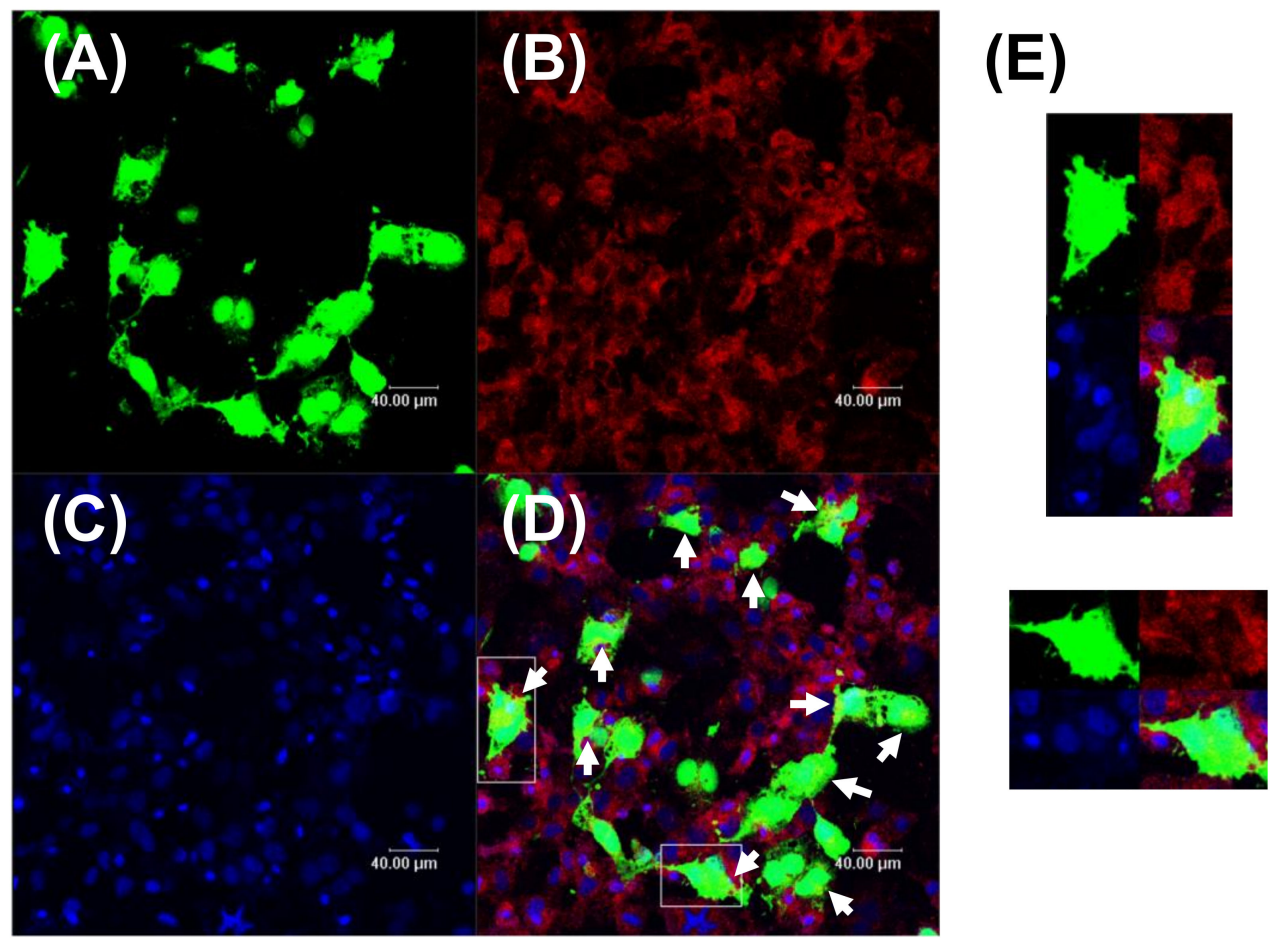
Confocal Laser Scanning Microscope (CLSM) images of MCF-7 cells transfected with DOX-NPs/pEGFP complexes at the optimal *w*/*w* ratio of 10. (**A**) Expressed EGFP fluorescence (green); (**B**) DOX fluorescence (red); (**C**) cell nuclei stained by DAPI (blue); (**D**) merged image of (A–C); (**E**) magnifications of the image in the white box. Scale bars in all images represent 40 μm.

The key point of co-administration is to deliver the drug and gene into the same cell and then achieve a synergistic therapeutic effect. Herein, the DOX-NPs/pEGFP complexes mediated co-localization of DOX, and expressed EGFP in MCF-7 cells that was directly visualized under confocal microscopy. Since the sample was washed three times with PBS before observation, the appeared fluorescence in the image was inside the cells, rather than outside the cells. Moreover, since the MCF-7 cells were cultured as a monolayer on the substrate, the overlapped fluorescence should arise from the same cell, not multiple cells. As shown in Figure 5, significant green GFP and red DOX signals were observed in MCF-7 cells, and yellow signals, representing their overlap, were also found in these cells (Figure 5D, at the site pointed by the arrows; Figure 5E). However, due to the strong signal of expressed GFP, most of the DOX and DAPI signals were masked by the green GFP. Similar phenomena were also found in Zhang and Lin’s studies [36,37]. To characterize the co-localization of gene and drug, the most used strategy is to stain both of gene and drug and observe their cellular uptake [38,39]. However, in our work, we directly observed the co-localization of the expressed protein and drug. This is more direct and precise than observation of cellular uptake of gene and drug. These results demonstrated that DOX-NPs/pEGFP complexes successfully transported DOX and pDNA into the same cell, and possessed high level of transfection capability.

### 2.5. Synergistic Anticancer Effect

**Figure 6:**
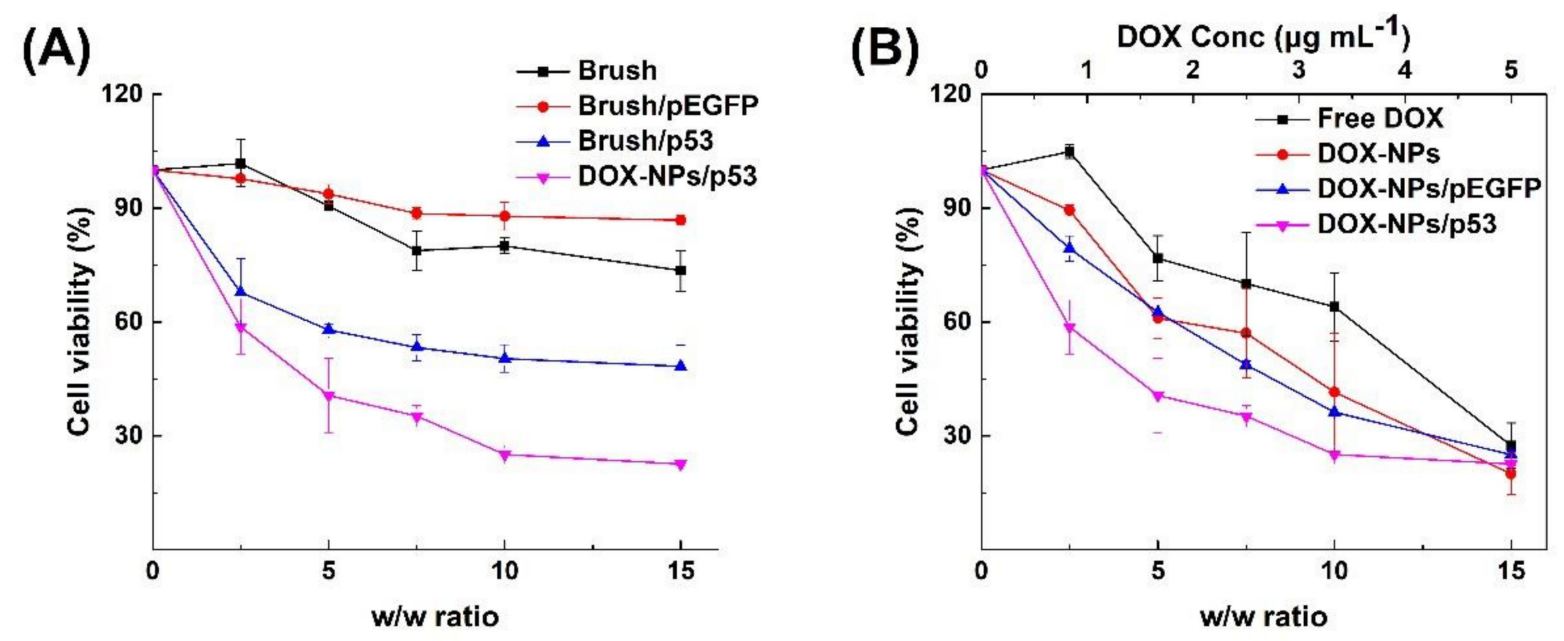
In vitro cytotoxicity against MCF-7 cells: (**A**) samples containing the polycationic brush (Brush, Brush/pEGFP, Brush/p53 and DOX-NPs/p53 complexes); and (**B**) samples containing DOX (free DOX, DOX-NPs, DOX-NPs/pEGFP and DOX-NPs/p53 complexes).

Based on the above co-delivery and transfection results, in vitro synergistic cancer therapeutic effects on MCF-7 cancer cells were then investigated by co-delivery of DOX and a p53-encoding plasmid. It is well known that p53 protein is a common tumor suppressor which has a pivotal role in cellular pathways, such as DNA repair, regulation of cell cycle, and cell apoptosis [40]. A mutant p53 gene loses the ability to control cell cycle and cell apoptosis, which results in tumor growth. Re-introduction of wild type p53 into tumor cells via effective carrier can re-stimulate cell apoptosis and then suppress cell proliferation. Moreover, stress signals in cells, such as DNA damage caused by drugs (e.g., DOX), can also induce a positive response of p53 [41], which would also contribute the inhibition of tumor growth. For this purpose, DOX-NP/p53 complexes were prepared, and their cytotoxicities on MCF-7 cancer cells were employed to evaluate the synergistic effect in vitro. Results in Figure 6A show the cytotoxicities of samples containing polycationic brushes (Brush, Brush/pEGFP, Brush/p53 and DOX-NPs/p53). As negative controls, the polycationic brush exhibited certain toxicity due to the positive charges of amino groups. When condensed with the pEGFP plasmid, the cytotoxicity of brush/pEGFP complexes decreased since the DNA neutralized some of the positive charges. However, when the p53 plasmid was introduced, the cell viability significantly decreased after incubated with brush/p53 complexes, which demonstrated the contribution of expressed p53 protein in cytotoxicity. Furthermore, when DOX and the p53 gene were simultaneously delivered to the same cell by DOX-NPs/p53 complexes, the cell growth was significantly inhibited compared to the ones with only p53 plasmid samples. Similar trends were also found in the samples that contained DOX. As shown in Figure 6B, at the same DOX concentration, DOX-NPs/pEGFP and DOX-NPs exhibited similar cytotoxicities towards MCF-7 cells, while the introduction of the p53 plasmid further decreased the cell viability. At *w*/*w* ratio of 10, the cell viability decreased to less than 30% after incubating with DOX-NPs/p53 complexes. However, when treated with only DOX, a greater DOX dosage was needed to achieve the similar antitumor effect. These results demonstrate that the polycationic brush could serve as an effective multifunctional carrier to load an appropriate dose of DOX and pDNA. They also show that synergistic anticancer effects with a lower DOX dosage can be achieved through co-delivery of DOX and a p53-encoding plasmid to the same cell. Generally, excessive chemotherapy would induce multi-drug resistance or enhanced toxicity and side effects [42]. Our results showed that the synergistic effect of DOX and the p53 gene decreased the DOX dosage and enhanced cell growth inhibition, which is beneficial to improve the quality of patient life. However, much remains to be investigated in the future. Different cells have different responses to the materials, the drugs and the genes, and these factors would influence the cellular uptake of the carriers, gene transfection efficiency and the synergistic effect of drugs and genes. Therefore, currently, many studies of co-delivery drug and gene from different groups focused on only one cell type [8,36,37,43,44]. It is somehow challenging to develop a co-delivery system that could work on more than one cell type. We consider that the mechanism of cell type dependence on the co-delivery system is worth conducting in detail, which would facilitate the development of broad-spectrum co-delivery systems.

## 3. Conclusions

In summary, a polycationic brush with “cyclodextrin-containing star polymers” as side chains was constructed to be a multifunctional carrier to co-deliver DOX and a gene. DOX-NPs were first prepared by host-guest interactions between CDs on the brush and hydrophobic DOX. A faster DOX release rate was achieved at pH 5.0 than 7.4, demonstrating DOX-NPs were able to release payload under endosomal- or lysosomal-pH. At the same time, pDNA was well condensed by DOX-NPs to form DOX-NPs/pDNA complexes with a size less than 150 nm. The complexes were able to transport DOX and a gene into the same cell, and had good transfection efficiency on MCF-7 cancer cells. As a result, enhanced cell growth inhibition with decreased DOX dosage was achieved due to the synergistic effect of co-delivery of DOX and a p53-encoding gene. This finding provides an efficient approach for the development of a co-delivery system in combination therapy.

## Figures and Tables

**Table 1:** Characteristics of DOX-NPs with different drug/polymer feed ratios.

Feed Ratio (Drug/Polymer, *w*/*w*)	LC (%)	EE (%)	D_h_ (nm)	PDI	Zeta Potential (mV)
2/10	5.0 ± 0.3	22.1 ± 1.0	61 ± 3	0.24 ± 0.02	6.6 ± 1.0
3/10	7.3 ± 0.3	23.0 ± 0.9	76 ± 3	0.38 ± 0.04	6.8 ± 1.0
5/10	21.5 ± 0.5	45.2 ± 1.0	190 ± 7	0.69 ± 0.04	9.6 ± 0.5

## Supplementary Materials

The following are available online at http://www.mdpi.com/2073-4360/11/1/60/s1, Materials and Methods, Figure S1: ^1^H NMR spectrum of the polycationic brush, Figure S2: GPC spectrum of polycationic brush.
