# Supplementary material for: A Polycationic Brush Mediated Co-Delivery of Doxorubicin and Gene for Combination Therapy
Source: Polymers (Basel). 2019 Jan 3;11(1):60. doi: 10.3390/polym11010060 (PMC6401996; doi:10.3390/polym11010060)
Supplement: Supplementary file 1 [file polymers-11-00060-s001.pdf]

Electronic Supplementary Information:

A polycationic brush mediated co-delivery of doxorubicin and gene for combination therapy

Wenjuan Chen [1], Mingming Zhang [1,*], Wei Shen [1], Bo Du [1], Jing Yang [1] and Qiqing Zhang [1,2,*]

[1] Tianjin Key Laboratory of Biomedical Materials, Institute of Biomedical Engineering, Chinese Academy of Medical Sciences & Peking Union Medical College, Tianjin 300192, P. R. China; chenwenjuanvv@163.com (W.C.); sw895971588@163.com (W.S.); dubojlu@163.com (B.D.); yangjing37@hotmail.com (J.Y.)

[2] Institute of Biomedical and Pharmaceutical Technology, Fuzhou University, Fuzhou 350002, P. R. China

* Correspondence: zhangmm@bme.cams.cn (M.Z.); zhangqiq@126.com (Q.Z.); Tel.: +86-22-87890868

S1. Materials and Methods

S1.1. Materials

The polycationic brush was synthesized according to our previous work [32]. Doxorubicin hydrochloride (DOX·HCl) was purchased from Dalian Meilun Biology Co., Ltd. (Dalian, China). The pEGFP-*C1* plasmid (Takara, kyoto, Japan) encoding enhanced green fluorescence protein (EGFP) and the p53-GFP plasmid (Addgene, Watertown, MA, USA) encoding p53 and GFP protein, were amplified in *Escherichia coli* and purified according to an Endo Free Plasmid Kit (Tiangen, Beijing, China). MCF-7 cells were gifts from School of Pharmacy, Tianjin Medical University. Dulbecco's modified Eagle medium (DMEM) and fetal bovine serum (FBS) were obtained from Thermo Scientific (Waltham, MA, USA). Phosphate buffered saline (PBS) and trypsine-EDTA solution were bought from Solarbio Technology Co., Ltd. (Beijing, China). The Cell Counting Kit-8 (CCK-8) was purchased from Dojindo Laboratories (Kumamoto, Japan).

S1.2. Preparation of DOX-Loaded Nanoparticles (DOX-NPs) via Host–Guest Interaction between CD Polymer and DOX

A series of DOX-NPs with different drug/polymer feed ratios were prepared by dialysis method. DOX·HCl and the polymer with various weight ratios were co-dissolved in DMSO at a polymer concentration of 10 mg mL^{-1}. Triethylamine was then added to remove hydrochloride. After stirring for 24 h, the mixtures were placed into dialysis bags (MWCO 7000) for dialyzing against deionized water at room temperature for 24 h. Then the dialysate was filtered through a 0.45 μm syringe filter and recovered by lyophilization.

S1.3. Preparation of DOX-NPs/pDNA Complexes

DOX-NPs and pDNA solutions were diluted in PBS solution (1 mM, pH 6.0) individually prior to being mixed together to obtain DOX-NPs/pDNA complexes at various N/P ratios. Complexes were formed by slowly dropping DOX-NPs solution into an equal volume of pDNA solution and incubating at room temperature for 30 min. Complexes were freshly prepared before each individual experiment.

S1.4. Characterization

The size, size distribution and zeta potential of DOX-NPs with various drug/polymer ratios and DOX-NPs/pDNA complexes with different N/P ratios in aqueous solution were determined by a Malvern Zetasizer Nano ZS instrument (Malvern Panalytical, Malvern, UK) at 25 °C. Atomic force microscopy (AFM) images were collected on a Nanoscope V atomic force microscope (Bruker, Billerica, MA, USA).

S1.5. Drug Loading

Drug loading content (LC) and encapsulation efficiency (EE) were determined by UV/Vis spectrometry (Pekin Elmer Lambda 35) using the following standard method. Freeze-dried DOX-NPs (5.0 mg) were dissolved in 1 mL of DMSO and diluted with 9 mL of water. The solution was measured by UV/Vis spectrometry at 480 nm, and the total amount of DOX was calculated from a standard calibration curve obtained from a series of DOX solutions in DMSO/H_2O (1/9, *v*/*v*). Each sample was analyzed in triplicate. LC and EE were calculated using Equations (1) and (2), respectively.

$$\mathrm{LC}\ (\%) = \frac{\text{weight of DOX in DOX} - \text{NPs}}{\text{weight of DOX} - \text{NPs}} \times 100\% \quad (1)$$

$$\mathrm{EE}\ (\%) = \frac{\text{weight of DOX in DOX} - \text{NPs}}{\text{weight of DOX feed initially}} \times 100\% \quad (2)$$

S1.6. In Vitro Release Study

The lyophilized DOX-NPs were dissolved in deionized water with a final DOX concentration of 1.0 mg mL^{-1}. The sample solution (4.0 mL) was transferred into one hole of a diffusion cell equipped with a dialysis membrane (MWCO 7000 Da), and 4 mL of phosphate buffer saline (PBS, 10 mM, pH 7.4) solution or acetate buffer saline (ABS, 10 mM, pH 5.0) solution were added into another hole of the cell. The diffusion cells were incubated in a shaker at 37°C. At predetermined time intervals, the test solution (1.0 mL) was withdrawn and replaced with an equal volume of the fresh medium. The DOX concentration in the release medium was determined using UV/Vis absorbance at 480 nm. All release experiments were carried out in triplicate.

S1.7. Gel Retardation Analysis

DOX-NPs/pDNA complexes (10 μL, containing 0.2 μg of pDNA) were mixed with 2 μL of loading buffer and loaded on a 0.8% agarose gel containing SYBR Green. Electrophoresis was performed in tris-acetate (TAE) running buffer at a voltage of 120 V for 0.5 h on a GE HE 33 system. DNA bands were visualized and photographed on a GE LAS 4500 system.

S1.8. In Vitro Transfection and Co-Delivery of DOX and pDNA

MCF-7 cells were seeded in the confocal dish at a density of 1 × 10^5 cells/dish and cultured in DMEM supplemented with 10% FBS for 24 h. At the time of transfection, the medium in each well was replaced with serum-free medium containing DOX-NPs/pEGFP complex solutions at *w*/*w* ratio of 10. After 4 h, the transfection medium was replaced with fresh DMEM containing 10% FBS. Following an additional 44 h, the supernatant was carefully removed and the cells were washed with PBS three times. The cells were fixed with 4% formaldehyde for 20 min and washed with PBS. Then 0.5 mL of DAPI (1 μg mL^{-1}) was added and incubated with the cells for 5 min to stain the nuclei of the cells. The cells were rinsed with PBS three times and the stained samples were observed by confocal laser scanning microscopy (CLSM) (Zeiss, LSM710, Oberkochen, Germany).

S1.9. Cell Viability

MCF-7 cells were seeded in 96-well plates at a density of 7000 cells/well. The cells were cultured in DMEM, supplemented with 10% FBS at 37 °C, under 5% CO_2. After incubated for 24 h, the culture media were replaced with fresh media containing brush, brush/pEGFP, brush/p53, DOX-NPs/p53, free DOX, DOX-NPs, and DOX-NPs/pEGFP complexes. The media were removed after 4 h, and replaced with fresh DMEM containing 10% FBS. After another incubation for 44 h, 10 μL of CCK-8 regents (Dojindo Laboratories, Kumamoto, Japan) and 90 μL of fresh media were added to each well. After 4 h, the plate was gently shaken for 2 min to dissolve any formazan crystals. The absorbance was measured using a multifunctional ELISA plate reader (Thermo Varioskan Flash, Waltham, MA, USA) at 450 nm. All experiments were carried out in triplicate. Cell viability was calculated according to Equation (3):

$$\text{Cell viability (\%)} = \frac{A_{sample} - A_{Blank}}{A_{Control} - A_{Blank}} \times 100\% \quad (3)$$

where A_{Sample} and $A_{Control}$ represent the absorbance of CCK-8 regents determined for cells treated with different samples and for control cells (untreated), respectively. A_{Blank} is the absorbance of CCK-8 regents without cells.

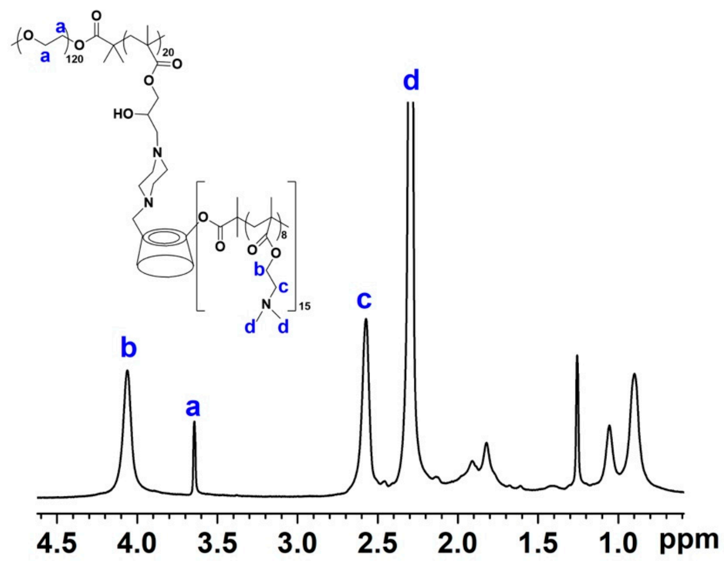

Figure S1. ^{1}H NMR spectrum of polycationic brush in $CDCl_3$.

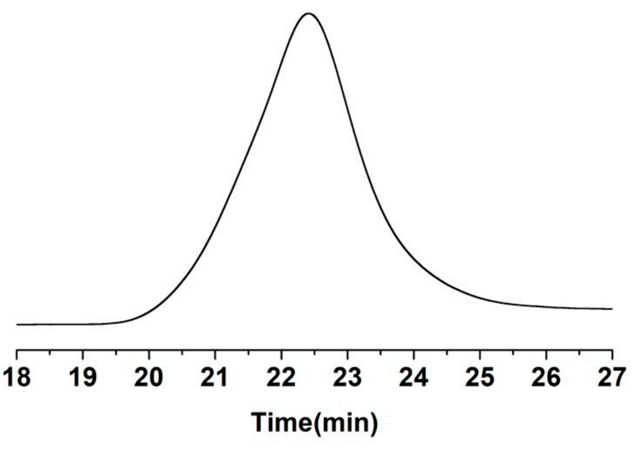

Figure S2. GPC spectrum of polycationic brush.

References:

1. Zhang, M.; Xiong, Q.; Wang, Y.; Zhang, Z.; Shen, W.; Liu, L.; Wang, Q.; Zhang, Q. A well-defined coil–comb polycationic brush with "star polymers" as side chains for gene delivery. *Polymer Chemistry* **2014**, *5*, 4670-4678, doi:10.1039/c4py00311j.
